# Author Correction: In vivo measurement of an Apelin gradient with a genetically encoded APLNR conformation biosensor

**DOI:** 10.1038/s41467-025-62537-9

**Published:** 2025-08-04

**Authors:** Lukas Herdt, Hannes Schihada, Michael Kurz, Sebastian Ernst, Jean Eberlein, Peter Kolb, Cornelius Krasel, Moritz Bünemann, Christian S. M. Helker

**Affiliations:** 1 Department of Biology, Animal Cell Biology, Marburg University, Marburg, Germany; 2 Institute of Pharmaceutical Chemistry, Faculty of Pharmacy, Marburg University, Marburg, Germany; 3 Institute of Pharmacology and Clinical Pharmacy, Faculty of Pharmacy, Marburg University, Marburg, Germany

**Keywords:** Biosensors, Development, Fluorescent proteins, G protein-coupled receptors

Correction to: *Nature Communications* 10.1038/s41467-025-61781-3, published online 21 July 2025

In this article, a panel in Figure 4b was inadvertently duplicated.

Original figure 4:

**Figure:**
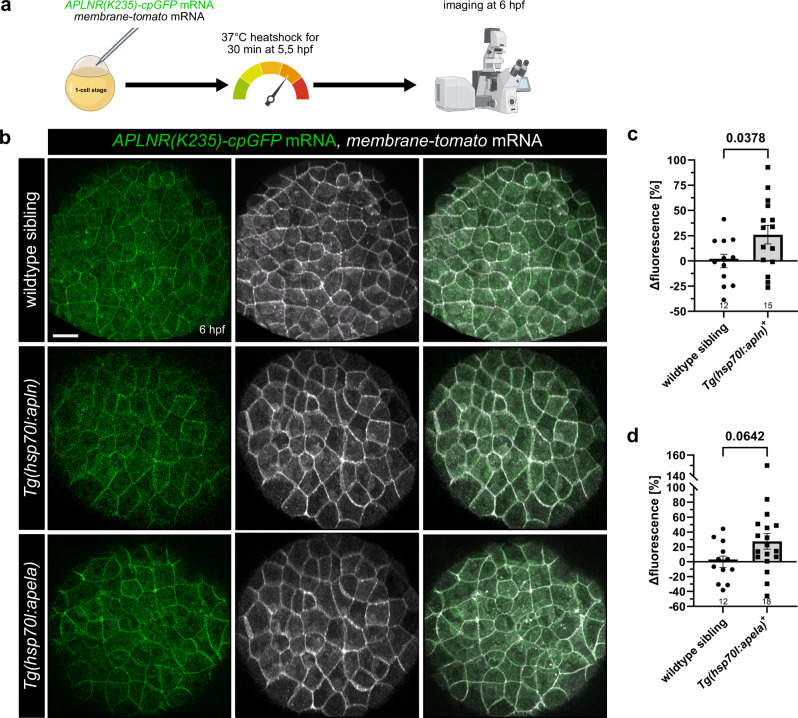

Corrected figure 4:

**Figure:**
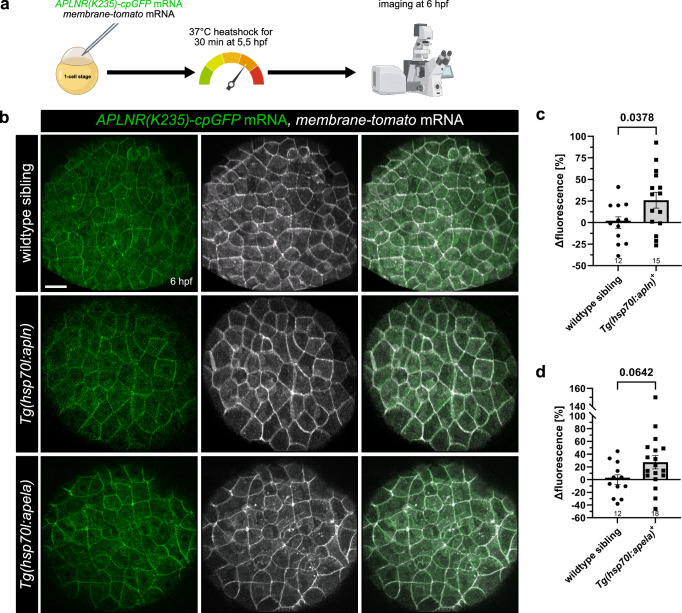

The original article has been corrected.

