# Supplementary material for: In vivo measurement of an Apelin gradient with a genetically encoded APLNR conformation biosensor
Source: Nat Commun. 2025 Jul 21;16:6682. doi: 10.1038/s41467-025-61781-3 (PMC12280157; doi:10.1038/s41467-025-61781-3)
Supplement: Supplementary file 1 — Supplementary Information [file 41467_2025_61781_MOESM1_ESM.pdf]

In vivo measurement of an Apelin gradient with a genetically encoded APLNR conformation biosensor

Lukas Herdt[†,1], Hannes Schihada[†,2], Michael Kurz[3], Sebastian Ernst[3], Jean Eberlein[1], Peter Kolb[2], Cornelius Krasel[3], Moritz Bünemann[3], Christian S.M. Helker[1,*]

1. Marburg University, Department of Biology, Animal Cell Biology, Karl-von-Frisch-Straße 8, 35043 Marburg, Germany

2. Marburg University, Faculty of Pharmacy, Institute of Pharmaceutical chemistry, Marbacher Weg 6-10, 35037 Marburg, Germany

3. Marburg University, Faculty Pharmacy, Institute of Pharmacology and clinical pharmacy, Karl-von-Frisch-Straße 2, 35043 Marburg, Germany

[†]Authors contribute equally

*Corresponding author: christian.helker@biologie.uni-marburg.de

Supplementary Figure 1-5

Supplementary Table 1-4

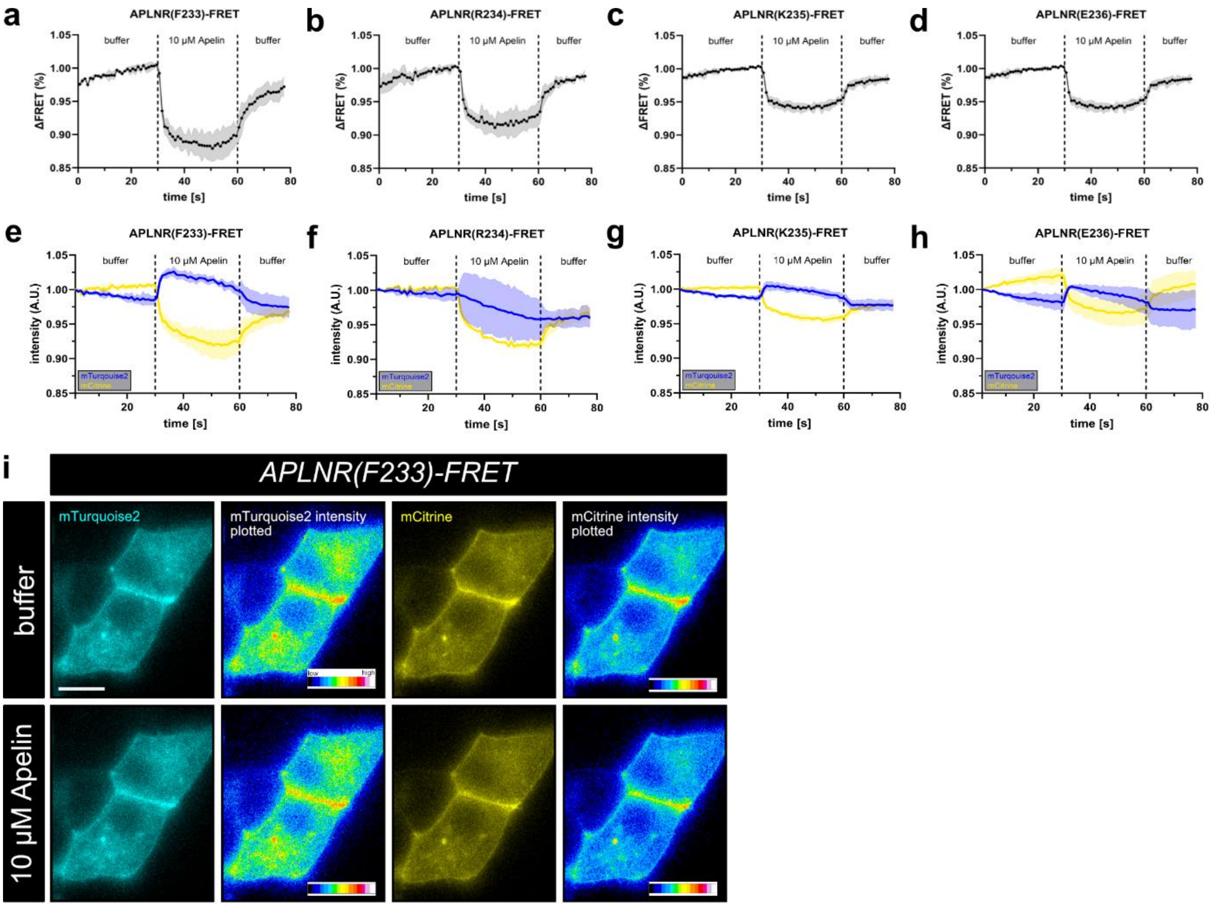

Supplementary Fig. 1. Real-time measurement of APLNR-FRET biosensors. (a-d) Stimulation of different APLNR-FRET variants (F233, R234, K235 and E236, which are the mCitrine integration sites in the APLNR) with buffer or 10 µM Apelin. Apelin superfusion led to a decrease in the FRET ratio of all four biosensor, which is reversible when superfused with buffer again. **(e-h)** Intensity traces of mTurquoise and mCitrine emission detected at 480 nm and 535 nm, respectively (excited at 425 nm), of the different APLNR-FRET biosensors stimulated with buffer or 10 µM Apelin. mTurquoise2 and mCitrine emission are corrected for background intensity, bleed through and false excitation. (**i**) Representative image of the APLNR(233)-FRET biosensor superfused with buffer or 10 µM Apelin. Data are represented as mean ± StD from three transiently transfected single HEK293T cells. Scale bars 10 µm. FRET - Förster resonance energy transfer; HEK293 – human embryonic kidney 293. Source data are provided as a Source Data file.

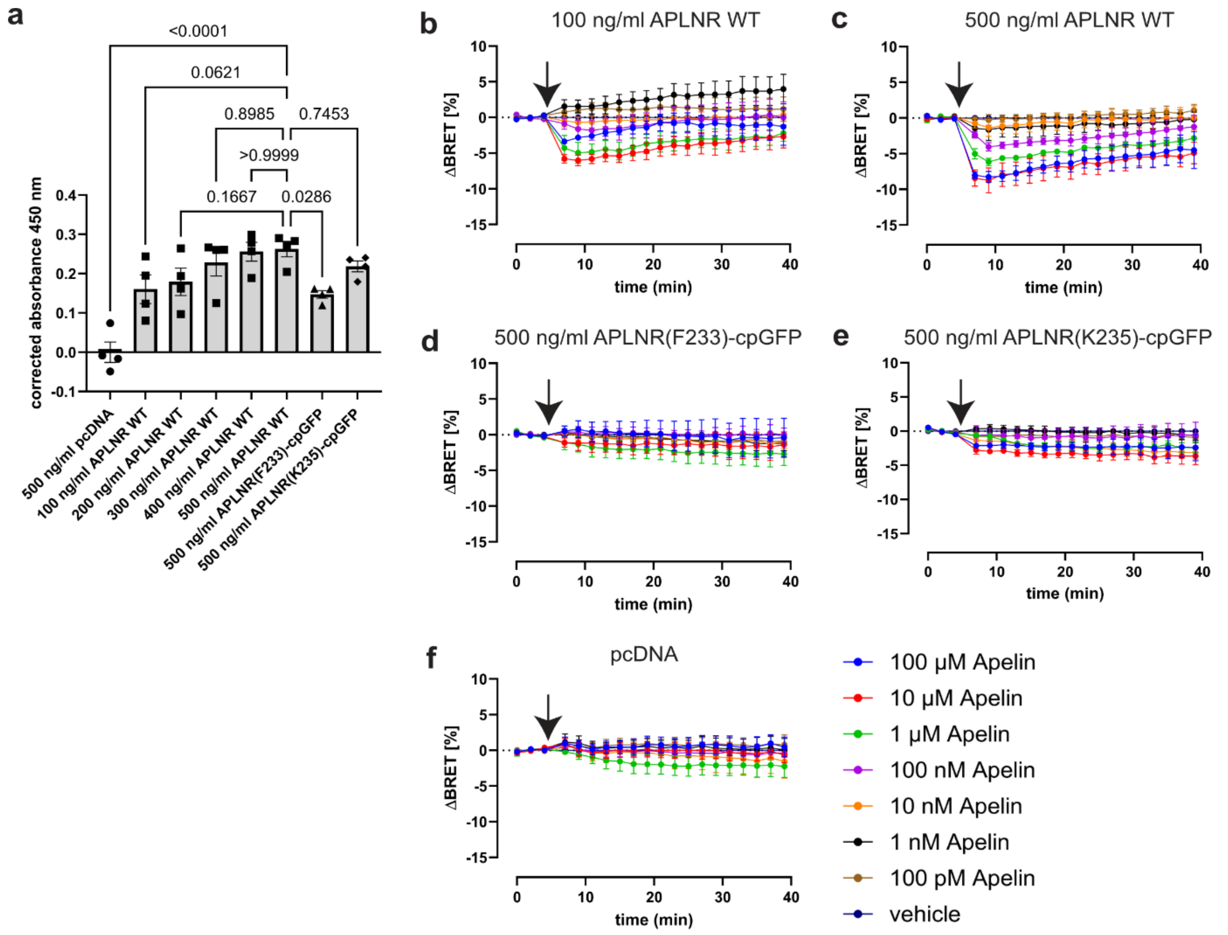

Supplementary Fig. 2. APLNR-dependent G_{i1} activation upon Apelin stimulation. (**a**) Surface expression levels of APLNR WT, APLNR(F233)-cpGFP and APLNR(K235)-cpGFP biosensors were quantified using ELISA against N-terminal HA tag. Different amounts of plasmid DNA encoding for the APLNR WT was used for transfection in HEK293A cells (ng plasmid DNA/ml transfected cells) to adjust APLNR WT expression levels to the APLNR-cpGFP biosensor variants. (**b-f**) ΔBRET time courses of the G_{i1} BRET sensor upon stimulation with various Apelin ligand concentrations. G_{i1} BRET sensor was co-transfected with either APLNR WT (**b-c**), APLNR(F233)-cpGFP (**d**), APLNR(K235)-cpGFP (**e**) or an empty pcDNA vector (**f**). The amounts of transfected DNA of the receptor plasmid (ng per ml of cells) was adapted based on the ELISA experiments (**a**) to ensure comparable surface protein amounts. Arrow indicates the timepoint of ligand addition. Data are presented as mean ± SEM from three independent experiments conducted in transiently transfected HEK293A cells. BRET - bioluminescence resonance energy transfer; cpGFP – circularly permuted GFP; WT - wildtype; HEK293 – human embryonic kidney 293. Source data are provided as a Source Data file.

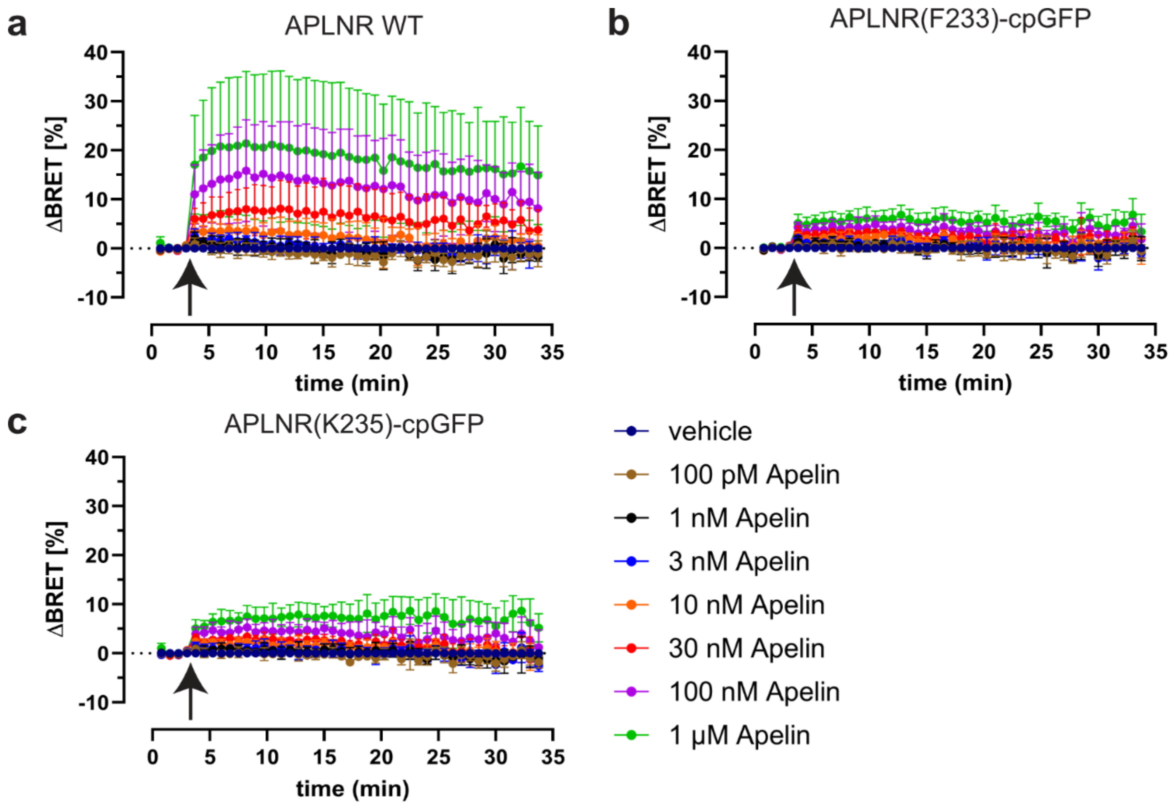

Supplementary Fig. 3. APLNR-dependent ARR3 recruitment upon Apelin stimulation. (**a-c**) ΔBRET time courses of the ARR3-BRET sensor upon stimulation with various Apelin ligand concentrations. ARR3-BRET sensor was co-transfected with either APLNR WT (**a**), APLNR(F233)-cpGFP (**b**) or APLNR(K235)-cpGFP (**c**). Arrow indicates the timepoint of ligand addition. Data are presented as mean ± StD from four independent experiments conducted in transiently transfected HEK293T cells. BRET - bioluminescence resonance energy transfer; cpGFP – circularly permuted GFP; WT - wildtype; HEK293 – human embryonic kidney 293. Source data are provided as a Source Data file.

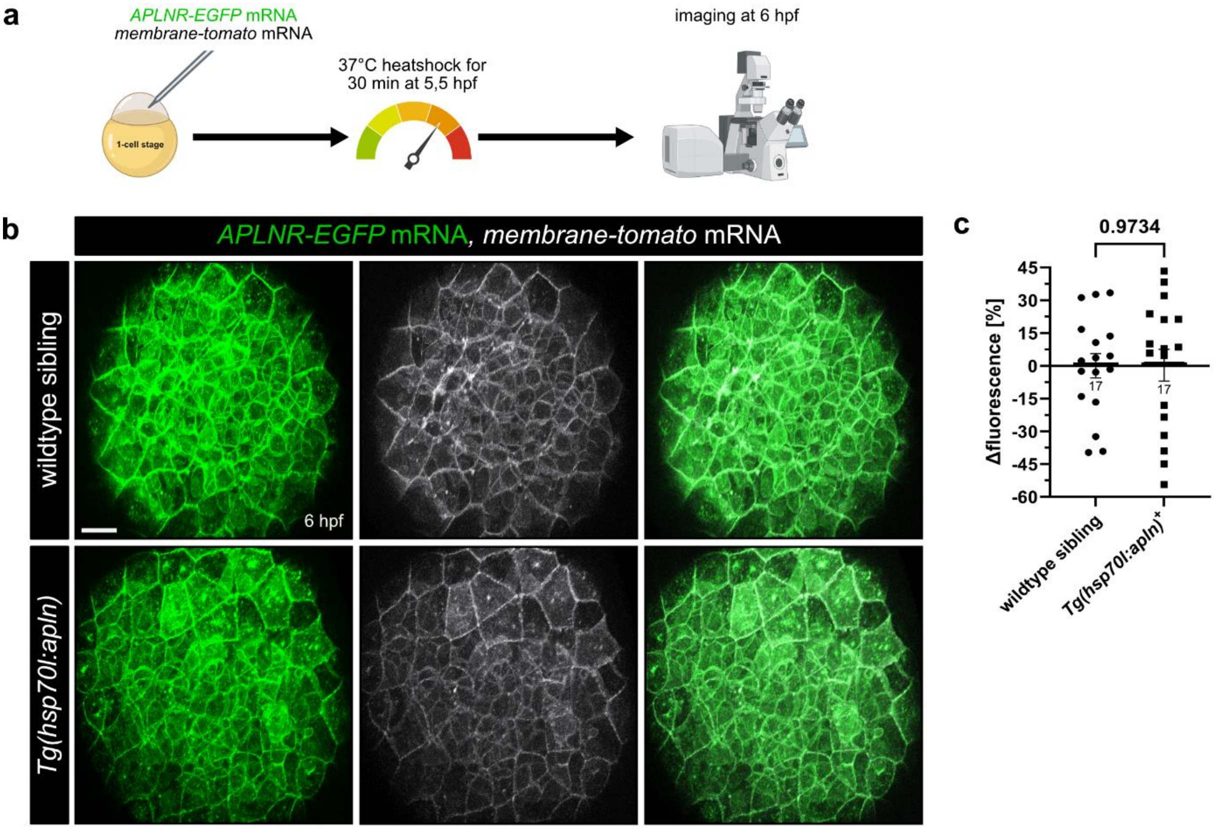

Supplementary Fig. 4. Ubiquitous Apelin overexpression does not change APLNR-EGFP intensity. (**a**) Schematic illustration of the procedure. *APLNR-EGFP* and *membrane-tomato* mRNA were injected into 1-cell stage zebrafish embryos. At 5,5 hours post fertilization (hpf) injected embryos were heat shocked for 30 min at 37°C to induce ubiquitous ligand overexpression and subsequently imaged at 6 hpf. (**b**) Representative confocal projection images of an injected wildtype sibling and transgenic *Tg(hsp70l:apln)* embryo at 6 hpf. (**c**) Quantification of APLNR-EGFP delta fluorescence intensity of wildtype siblings compared to embryos ubiquitously overexpressing the *apln* ligand. Each dot represents the mean of 10 measured cells per embryo. Data are presented as mean values ± SEM. (N = number of embryos, n = number of cells: (**c**) wildtype siblings N/n: 17/170, *Tg(hsp70l:apln)*+ N/n: 17/170). Statistical analysis was performed by using two-tailed unpaired Student's t-test with Welch's correction. Scale bars 30 µm. Source data are provided as a Source Data file. (**a**) Created in BioRender. Schihada, H. (2025) https://BioRender.com/0kt43lb

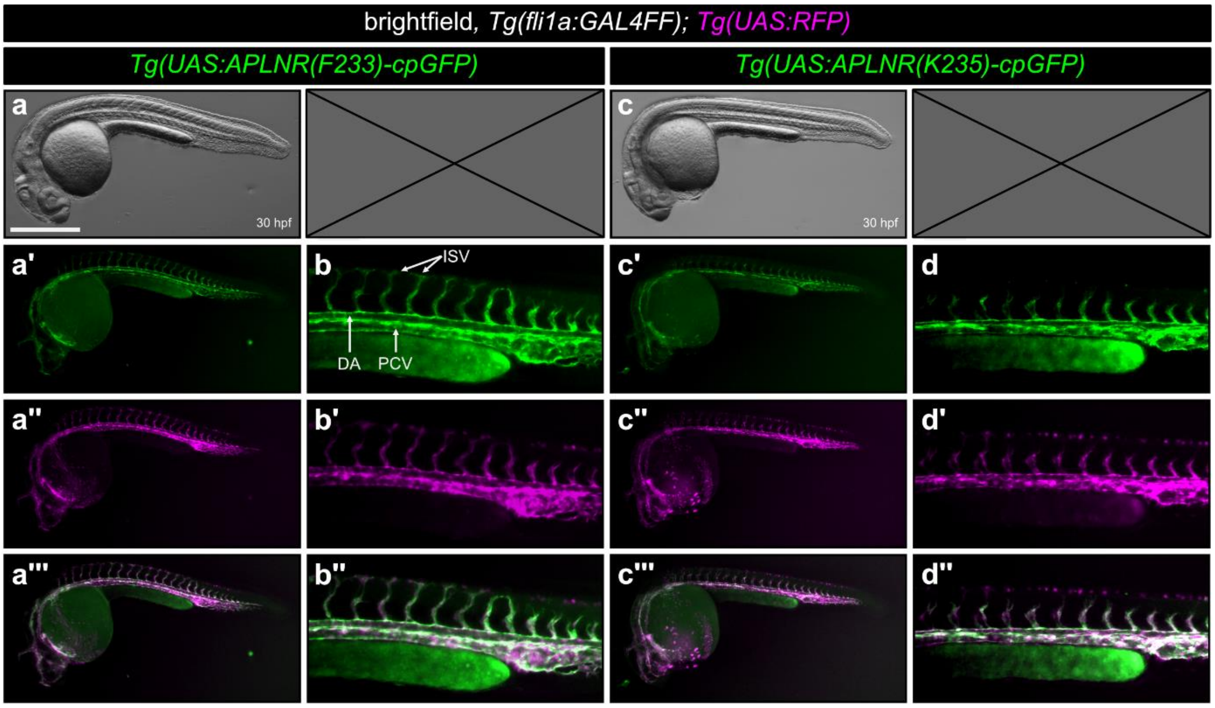

Supplementary Fig. 5. Vascular-specific expression of the APLNR-cpGFP biosensors. (a, c) Brightfield images of APLNR(F233)-cpGFP (**a**) and APLNR(K235)-cpGFP (**c**) biosensor embryos in a lateral view at 30 hpf. (**a-b**) Lateral views of *Tg(fli1a:GAL4FF); Tg(UAS:RFP); Tg(UAS:APLNR(F233)-cpGFP)* embryos of the whole embryo (**a'-a'''**) and of the trunk (**b'-b'''**). (**c-d**) Lateral views of *Tg(fli1a:GAL4FF); Tg(UAS:RFP); Tg(UAS:APLNR(K235)-cpGFP)* embryos of the whole embryo (**c'-c'''**) and of the trunk (**d'-d'''**). ISV – intersegmental vessel; DA – dorsal aorta; PCV – posterior cardinal vein. Scale bars 500 µm.

Supplementary Table 1. In vitro cloning primer

homology arm: capital letters

restriction site: small letters

construct-specific part: capital letters, underlined, bold

Primer name	Primer sequence (5‘ → 3‘)
APLNR se	CTAGCGCTTAAGGCCTGTTAaccggt**ACCGCCATGGAGGAAGGTGGTGATTTTG**
APLNR-mTurq2 as	CTGATCAGCGGTTTAAACTTaagctt**TTACTTGTACAGCTCGTCCATGC**
APLNR(F233) as	CTGATCAGCGGTTTAAACTTaagcttctcgagggatcc**GAAGTGGCCAGCGATGG**
APLNR(F233)-mCitrine se	AAACCATCGCTGGCCACTTCggatcc**GTGAGCAAGGGCGAGGAG**
APLNR(F233)-mCitrine as	CTGATCAGCGGTTTAAACTTaagcttctcgag**CTTGTACAGCTCGTCCATGCC**
APLNR(F233)-mTurq2 se	GCATGGACGAGCTGTACAAGctcgag**CGCAAGGAACGCATCGAG**
APLNR(R234) as	CTGATCAGCGGTTTAAACTTaagcttctcgagggatcc**GCGGAAGTGGCCAGC**
APLNR(R234)-mCitrine se	CCATCGCTGGCCACTTCCGCggatcc**GTGAGCAAGGGCGAGGAG**
APLNR(R234)-mCitrine as	CTGATCAGCGGTTTAAACTTaagcttctcgag**CTTGTACAGCTCGTCCATGCC**
APLNR(R234)-mTurq2 se	ATCACTCTCGGCATGGACGAGCTGTACAAGctcgag**AAGGAACGCATCGAGGGC**
APLNR(K235) as	CTGATCAGCGGTTTAAACTTaagcttctcgagggatcc**CTTGCGGAAGTGGCCAG**
APLNR(K235)-mCitrine se	TCGCTGGCCACTTCCGCAAGggatcc**GTGAGCAAGGGCGAGGAG**
APLNR(K235)-mCitrine as	CTGATCAGCGGTTTAAACTTaagcttctcgag**CTTGTACAGCTCGTCCATGCC**
APLNR(K235)-mTurq2 se	GCATGGACGAGCTGTACAAGctcgag**GAACGCATCGAGGGCC**
APLNR(E236) as	CTGATCAGCGGTTTAAACTTaagcttctcgagggatcc**TTCCTTGCGGAAGTGGCC**
APLNR(E236)-mCitrine se	CTGGCCACTTCCGCAAGGAAggatcc**GTGAGCAAGGGCGAGGAG**
APLNR(E236)-mCitrine as	CTGATCAGCGGTTTAAACTTaagcttctcgag**CTTGTACAGCTCGTCCATGCC**
APLNR(E236)-mTurq2 se	ATCACTCTCGGCATGGACGAGCTGTACAAGctcgag**CGCATCGAGGGCCTG**
APLNR-nLuc as	CTGATCAGCGGTTTAAACTTaagctt**TTACGCCAGAATGCGTTCG**
APLNR(F233)-nLuc se	GCATGGACGAGCTGTACAAGctcgag**CGCAAGGAACGCATCGAG**
APLNR(R234)-nLuc se	GCATGGACGAGCTGTACAAGctcgag**AAGGAACGCATCGAGGGC**
APLNR(K235)-nLuc se	GCATGGACGAGCTGTACAAGctcgag**GAACGCATCGAGGGCCT**
APLNR(E236)-nLuc se	GCATGGACGAGCTGTACAAGctcgag**CGCATCGAGGGCCTGC**
N-terminal-APLNR-cpGFP as	CTGATCAGCGGTTTAAACTTaagcttctcgag**CAGTTGGTCATGATTGTTGTACTCCA**
cpGFP-APLNR-C-terminal as	ACAGTCGAGGCTGATCAGCGGTTTAAACTTaagctt**CTAGTCAACCACAAGGGTCTCC**
N-terminal-APLNR(F233)-cpGFP se	AAACCATCGCTGGCCACTTCggatcc**CTGAGCTCACTCATTAACGTCTATATCAAG**

cpGFP-APLNR(F233)-C-terminal se	AAGCTGGAGTACAACAATCATGACCAACTGctcgag**CGCAAGGAACGCATCGAG**
N-terminal-APLNR(R234)-cpGFP se	CCATCGCTGGCCACTTCCGCggatcc**CTGAGCTCACTCATTAACGTCTATATCAAG**
cpGFP-APLNR(R234)-C-terminal se	AAGCTGGAGTACAACAATCATGACCAACTGctcgag**AAGGAACGCATCGAGGGC**
N-terminal-APLNR(K235)-cpGFP se	TCGCTGGCCACTTCCGCAAGggatcc**CTGAGCTCACTCATTAACGTCTATATCAAG**
cpGFP-APLNR(K235)-C-terminal se	AAGCTGGAGTACAACAATCATGACCAACTGctcgag**GAACGCATCGAGGGCC**
N-terminal-APLNR(E236)-cpGFP se	CTGGCCACTTCCGCAAGGAAggatcc**CTGAGCTCACTCATTAACGTCTATATCAAG**
cpGFP-APLNR(E236)-C-terminal se	AAGCTGGAGTACAACAATCATGACCAACTGctcgag**CGCATCGAGGGCCTG**
Puromycin se	TTTTGCAAAAAGCTcccggg**ATGACCGAGTACAAGCCCAC**
Puromycin as	GCTTGGTCGGTCATttcgaa**TCAGGCACCGGGCTTGCGGG**
HA-APLNR se	CTAGCGCTTAAGGCCTGTTAaccggt**GCCACCATGTACCCTTACGATGTACCGGATTACGCAGAGGAAGGTGGTGATTTTGACAAC**
mScarlet-I3 se	AGGAGACCCTTGTGGTTGACgaattc**GATAGCACCGAGGCAGTGATC**
p2A-mScarlet-I3 se	CAGCCAGGAGACCCTTGTGGTTGACgaattc**GGTTCTGGTGCTACTAATTTTTCTTTATTAAAACAAGCTGGTGATGTTGAAGAAAATCCTGGTCCTGATAGCACCGAGGCAGTGATC**
mScarlet-I3 as	GCTGATCAGCGGTTTAAACTTaagctt**TTAGGAGCCACCGGAGCC**
*Hind*III-SYFP2 se	AAAAAAaagctt**ATGGTGAGCAAGGGCGAGG**
SYFP2-CAAX-*Not*I as	AAAAAAgcggccgg**CTAGGAGAGCACACACTTGCAGCTCATGCAGCCCGGGCCACTCTCATCAGGAGGGTTCTTGTACAGCTCGTCCATGC**

Supplementary Table 2. In vivo cloning primer

homology arm: capital letters

restriction site: small letters

construct-specific part: capital letters, underlined, bold

Primer name	Primer sequence (5‘ —> 3‘)
UAS-APLNR-cpGFP se	TTCGAATTAGATCTGTCGACgaattc**ACCGCCATGGAGGAAGG**
UAS-APLNR-cpGFP as	CGACTCACTATAGTTCTAGAGGctcgag**CTAGTCAACCACAAGGGTCTCC**
APLNR-cpGFP-pCS2+ se	CAAGCTACTTGTTCTTTTTGCAggatcc**ACCGCCATGGAGGAAGG**
APLNR-cpGFP-pCS2+ as	TTCTAGAGGCTCGAGAGGCCTTgaattc**CTAGTCAACCACAAGGGTCTCC**
pcDNA3.1 as	TAGAGGCTCGAGAGGCCTTgaattc**CTGATCAGCGGTTTAAACTTAAGCTT**
mScarlet-I3 as	ACTCACTATAGTTCTAGAGGctcgag**TTAGGAGCCACCGGAGCC**

Supplementary Table 3. Zebrafish genotyping primer

Primer name	Primer sequence (5‘ —> 3‘)
hsp70l se	CATGTGGACTGCCTATGTTCATC
apln as	CAGCTCGAGCGGGATTCT
apela as	GTTTCTTCGGGCAGTTGTGT

Supplementary Table 4. *apln* and *apela* crRNAs

crRNA target	crRNA sequence (5‘ —> 3‘)
apln crRNA #1	CTATGCTCGGTGGAGGCCAT
apln crRNA #2	GAATGTGAAGATCTTGACGC
apln crRNA #3	GAAGCATGAGGACTCCTTTG
apela crRNA #1	TGAGATTCTTCCACCCGCTG
apela crRNA #2	TCCAGAGTACCTTTCCCTTG
apela crRNA #3	GAAGAAACGCTGTCTACCTC
